# Scrotal elephantiasis secondary to hidradenitis suppurativa: a rare case report

**DOI:** 10.3389/fmed.2026.1867933

**Published:** 2026-06-03

**Authors:** Salem K. Qupp, Iyad M. Halayqa, Mohammad M. Zeidan, Layth Al-Karaja, Majed F. I. Alzeer Alhousseini

**Affiliations:** 1 Faculty of Medicine, Al-Quds University, Jerusalem, Palestine; 2 Rafidia Governmental Hospital, Rafidia, Palestine

**Keywords:** dermatology, hidradenitis suppurativa, lymphedema, scrotal edema, scrotal elephantiasis

## Abstract

**Background:**

Genital elephantiasis is a rare but debilitating condition characterized by progressive enlargement due to chronic lymphatic dysfunction. While most commonly associated with filarial infection, secondary causes include malignancy, trauma, surgery, and chronic inflammatory conditions. Scrotal involvement secondary to hidradenitis suppurativa is exceedingly rare.

**Case presentation:**

We report a 34-year-old male with long-standing, treatment-refractory hidradenitis suppurativa involving the groin, axilla, and chest, presenting with progressive massive scrotal enlargement over several years. Physical examination revealed a firm, non-tender scrotal mass measuring 20 × 16 × 6 cm. Laboratory investigations were within normal limits. The patient underwent complete surgical excision of the scrotal mass via en bloc resection with preservation of both testes and spermatic cords, followed by reconstruction with split-thickness skin grafting of the penile shaft. The resected specimen weighed approximately 6 kg. Histopathology demonstrated reactive fibromyxoid changes without evidence of malignancy.

**Conclusion:**

Scrotal elephantiasis secondary to hidradenitis suppurativa is an exceptionally rare complication reflecting advanced lymphatic destruction. Early recognition and timely surgical intervention are essential to restore function and quality of life. This case highlights that even biologic therapy may not prevent progression to end-stage lymphatic disease in refractory HS.

## Introduction

Genital elephantiasis is an uncommon condition outside regions where filariasis is endemic, yet it is associated with significant physical disability and psychological burden. Primary (congenital) lymphedema results from developmental abnormalities of the lymphatic system and is rare, as seen in conditions such as Meige disease. In contrast, secondary (acquired) chronic genital lymphedema may arise from a variety of causes, including infections such as lymphogranuloma venereum, filarial infestation due to Wuchereria bancrofti, recurrent inflammation, malignancy, lymph node dissection, trauma, or radiation exposure (1–3).

Hidradenitis suppurativa (HS) is a chronic, relapsing inflammatory disorder of the follicular–apocrine unit, characterized by painful nodules, abscesses, sinus tract formation, and scarring. The groin and anogenital regions are particularly prone to persistent inflammation, which may compromise lymphatic drainage (4). Although HS affects approximately 1% of the population, the development of secondary lymphedema and scrotal elephantiasis remains exceedingly rare, with only a limited number of cases reported in the literature (5, 6).

We report a rare case of massive scrotal elephantiasis secondary to long-standing, treatment-refractory hidradenitis suppurativa involving the groin, presenting with severe functional impairment and managed successfully through en bloc surgical excision and reconstructive repair with preservation of genital function.

## Case presentation

A 34-year-old male presented to the Urology Department in October 2023 with progressive, painless scrotal enlargement evolving over several years. He reported no systemic symptoms, including fever, night sweats, or weight loss. His past medical history was significant for severe hidradenitis suppurativa involving the axillae, inframammary region, and groin, consistent with Hurley stage III disease with extensive sinus tract formation, scarring, and recurrent abscesses. Overall disease severity was consistent with a high International Hidradenitis Suppurativa Severity Score System (IHS4) indicative of severe active disease.

The patient had been treated with multiple medical regimens, including prolonged systemic antibiotics and biologic therapy with adalimumab. Adalimumab was administered at the standard dosing regimen of 40 mg subcutaneously weekly following the induction phase and had been initiated approximately 14 months prior to presentation. Despite initial partial improvement, the response was not sustained, and the patient continued to develop recurrent inflammatory lesions in the axillary and groin regions, indicating ongoing active disease rather than complete remission at the time of presentation.

At the time of admission, although the scrotal disease had progressed to end-stage fibrotic lymphedema with no active acute inflammation locally, the patient still demonstrated intermittent inflammatory HS activity in other anatomical sites, particularly the axillae. Thus, his condition represented chronic, partially controlled but overall refractory HS rather than clinical remission.

**Figure 1:**
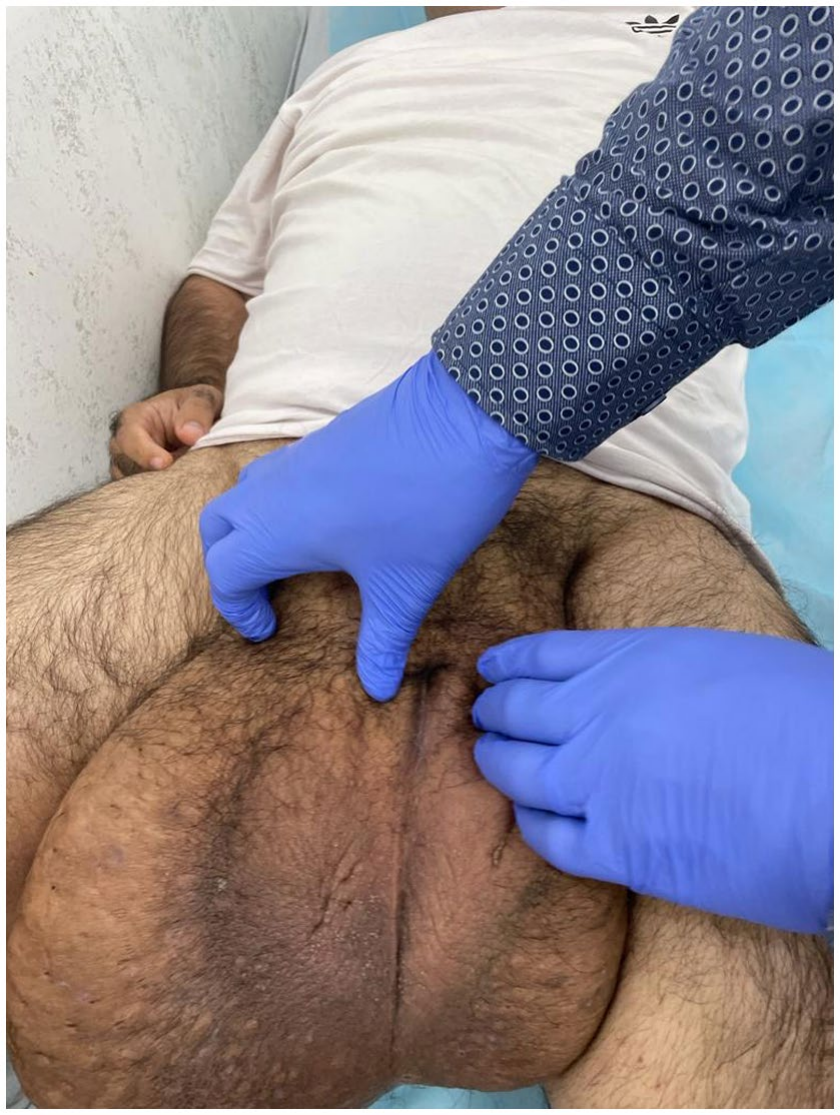
Preoperative clinical appearance showing marked enlargement of the scrotum with firm, non-tender consistency consistent with massive scrotal elephantiasis secondary to chronic lymphatic obstruction.

On physical examination, the scrotum was markedly enlarged, firm, and non-tender, measuring approximately 20 × 16 × 6 cm (Figure 1). No systemic abnormalities were identified on general examination.

Laboratory investigations were performed and returned entirely within normal limits, as summarized in Table 1. Complete blood count revealed a white blood cell count of 7.8 × 10^9^/L, hemoglobin of 17.2 g/dL, mean corpuscular volume of 82.2 fL, and platelets of 283 × 10^9^/L. Renal function and electrolytes were unremarkable, with a creatinine of 0.93 mg/dL, sodium of 139 mmol/L, and potassium of 4.3 mmol/L. Inflammatory markers showed a C-reactive protein of 0.62 mg/L. Coagulation studies were within normal range, with a prothrombin time of 13.2 s, an INR of 1.02, and an activated partial thromboplastin time of 29 s.

**Table 1:** Laboratory investigations.

Test	Result	Reference range
WBC (×10^9^/L)	7.8	4.6–11
Hemoglobin (g/dL)	17.2	13.5–17
MCV (fL)	82.2	80–100
Platelets (×10^9^/L)	283	150–450
Creatinine (mg/dL)	0.93	0.72–1.25
Sodium (mmol/L)	139	135–145
Potassium (mmol/L)	4.3	3.5–5.3
CRP (mg/L)	0.62	0–5
PT (sec)	13.2	11–15
INR	1.02	—
aPTT (sec)	29	25–35

Given the significant size of the mass and its functional and psychosocial impact, surgical excision was planned. The patient was admitted for surgical management. Under general anesthesia, he was placed in the lithotomy position. An 18 Fr two-way Foley catheter was inserted for urinary decompression. Preoperative marking of the planned incision lines was performed to guide both excision and reconstruction.

A scrotal incision was made using electrocautery, and dissection proceeded in an avascular plane between the scrotal skin and the underlying diseased tissue, with progressive mobilization of the mass from surrounding structures. Both testes were identified intraoperatively and carefully dissected free, with preservation of the spermatic cords and neurovascular supply bilaterally.

A circumferential incision at the penile base was then performed, allowing en bloc excision of the entire scrotal mass. The specimen measured 20 × 16 × 6 cm and weighed approximately 6 kg. Meticulous hemostasis was achieved throughout the procedure.

For reconstruction, partial excision of suprapubic subcutaneous fat was performed to facilitate tissue mobilization and contouring. The scrotal and perineal defect was closed. Penile resurfacing was performed using a split-thickness skin graft applied to the penile shaft, achieving adequate coverage and preservation of penile length and function.

Closure was performed in layers using absorbable Vicryl sutures for the subcutaneous tissue, and skin clips where applicable. A sterile dressing was applied, and the patient was transferred to the ward in stable condition.

Histopathological analysis was performed. Macroscopic examination of the specimen described a mass measuring 21 × 19 × 11 cm, covered by skin of corresponding dimensions, with additional fragments of fibrofatty tissue measuring 15 × 11 cm in aggregate, submitted in the same container. Step-sectioning of the mass revealed a myxoid cut surface throughout. Microscopic examination confirmed a diagnosis of reactive fibromyxoid changes on scrotal biopsy, with no evidence of malignancy.

**Figure 2:**
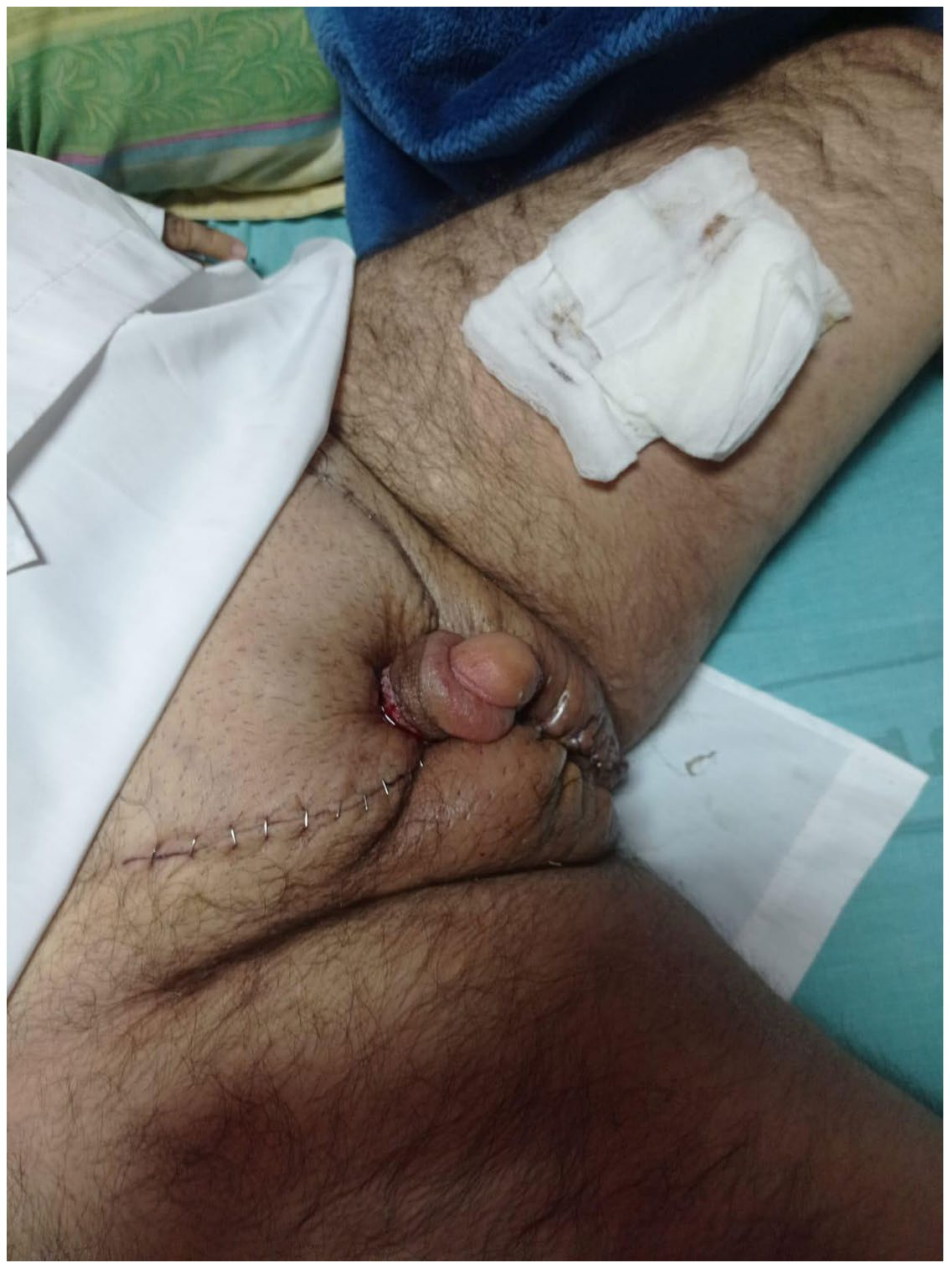
Postoperative follow-up demonstrating well-healed surgical site with satisfactory cosmetic outcome, preserved genital anatomy, and no evidence of recurrence.

The patient’s postoperative course was uneventful. Follow-up demonstrated excellent wound healing, preserved sexual function, and a satisfactory cosmetic outcome (Figure 2).

## Discussion

Hidradenitis suppurativa (HS) is a chronic inflammatory disorder of the follicular–apocrine unit, commonly affecting intertriginous areas such as the groin and anogenital regions (1). Its pathogenesis is multifactorial, involving genetic, hormonal, microbial, mechanical, and lifestyle factors such as obesity and smoking. Emerging evidence highlights keratinocytes as active contributors to disease pathogenesis, with intrinsic abnormalities in differentiation and inflammatory signaling that promote follicular hyperkeratosis, immune cell recruitment, and sustained local inflammation (7). Dysregulated innate and adaptive immune responses, including activation of macrophages, neutrophils, and proinflammatory cytokines such as IL-1β and tumor necrosis factor, together with Th1/Th17-mediated signaling, drive follicular destruction, sinus tract formation, fibrosis, and irreversible scarring (8, 9). In advanced disease, chronic inflammation and recurrent tissue injury may impair lymphatic drainage, leading to progressive dermal thickening, secondary lymphedema, and, in rare cases, massive scrotal elephantiasis (5, 10).

Management of hidradenitis suppurativa (HS) requires a stage-based, multidisciplinary approach combining medical, surgical, and supportive interventions tailored to disease severity and phenotype. Topical therapies, including clindamycin and resorcinol, may benefit patients with mild or localized disease, while systemic treatments such as oral antibiotics and biologic agents, including adalimumab, secukinumab, and bimekizumab, are indicated for inflammatory and moderate-to-severe disease to reduce disease activity and prevent progression. Surgical interventions, ranging from incision and drainage to deroofing and wide excision, remain essential for managing persistent abscesses, sinus tracts, and irreversible fibrotic changes, often in combination with systemic therapy. Adjunctive measures such as weight reduction, smoking cessation, friction avoidance, wound care, pain management, and treatment of associated comorbidities, including psychological support, are critical components of comprehensive care aimed at improving long-term outcomes and quality of life (8).

In our patient, longstanding HS refractory to adalimumab led to progressive lymphatic compromise and the development of scrotal elephantiasis. Histopathology confirmed a reactive fibro-myxoma, reflecting the chronic proliferative changes induced by longstanding lymphedema and inflammation. This demonstrates that even with biologic therapy, persistent HS may result in severe secondary complications.

Surgical excision remains the primary treatment for advanced scrotal elephantiasis, aiming to relieve discomfort, restore genital anatomy, preserve testicular and penile function, and achieve satisfactory cosmetic outcomes (3). Various reconstructive strategies exist, including mobilization of suprapubic skin flaps, staged excision with split-thickness skin grafts, and direct closure. In this case, partial resection of suprapubic subcutaneous tissue allowed mobilization for optimal reconstruction. Careful preservation of testicular structures and vascular supply was essential to maintain sexual and reproductive function.

Several case reports have documented scrotal elephantiasis or lymphedema as a rare but devastating complication of long-standing HS. Alharbi et al. (11) reported a 38-year-old man with scroto-perineal HS complicated by giant scrotal elephantiasis, successfully managed with wide local excision and split-thickness skin grafting, which preserved testicular and penile structures and achieved satisfactory outcomes. Good et al. (6) described a 58-year-old man with a decade-long history of HS who developed severe scrotal elephantiasis with draining sinus tracts; extensive evaluation excluded filariasis and other secondary causes, confirming lymphatic scarring from HS as the etiology, and the authors highlighted the profound psychosocial burden of this complication. More recently, Yu et al. (5) presented a 41-year-old man with massive scrotal lymphedema due to HS, treated with a modified Charles’ procedure involving complete excision while retaining the scrotal septum and subcutaneous lymphatic tissue flaps, resulting in restoration of function, preserved sensation, and excellent cosmetic results with no recurrence at 6 months. Collectively, these reports underscore that HS-associated scrotal lymphedema is uncommon but highly morbid, often requiring surgical intervention for both functional rehabilitation and psychosocial recovery.

This case underscores several key points: early recognition of lymphatic complications in HS patients, a multidisciplinary approach involving dermatologists, urologists, and reconstructive surgeons, and individualized surgical planning to optimize functional and cosmetic outcomes. Even with biologic therapy, chronic HS may lead to severe complications, highlighting the need for long-term vigilance.

## Conclusion

Massive scrotal elephantiasis secondary to hidradenitis suppurativa represents an uncommon but severe end-stage manifestation of chronic lymphatic injury resulting from prolonged inflammation and fibrosis. Despite advances in medical therapy, including biologics, refractory disease can progress to irreversible structural damage requiring radical surgical management.

This case emphasizes the importance of early multidisciplinary management of hidradenitis suppurativa to prevent advanced complications. When elephantiasis develops, complete surgical excision with carefully planned reconstruction remains the definitive treatment, offering effective restoration of anatomy, preservation of genital function, and significant improvement in quality of life.

## Data Availability

The original contributions presented in the study are included in the article/supplementary material, further inquiries can be directed to the corresponding author.
